# Klippel-Trenaunay Syndrome in the Distal Part of the Unilateral Upper Limb and Venous Deficiency: A Case Report

**DOI:** 10.31729/jnma.8892

**Published:** 2025-02-28

**Authors:** Beenu Maharjan, Lian Liu, Xu Liu, Qingfeng Liu, Xian Jiang

**Affiliations:** 1 Department of Dermatology, West China Hospital, Sichuan University, Chengdu 610041, China

**Keywords:** *case reports*, *Klippel-Trenaunay syndrome*, *port-wine stain*

## Abstract

Klippel-Trenaunay Syndrome is a rare congenital disorder characterized by a triad of symptoms-capillary malformations, venous abnormalities, and hypertrophy of bones and soft tissues. The syndrome often presents with port wine stains, varicose veins, and limb hypertrophy, which can lead to significant complications such as venous thromboembolism and bleeding. We present a case of a 13 ' year female, with erythema in left upper limb and shoulder since birth.. An effective treatment method was employed, which incorporated a multidisciplinary team of experts specialized in various fields of dermatology and vascular surgery, all working collaboratively with the patient's well-being at the core of their efforts. This approach emphasized a personalized strategy based on the patient's preferences.

## INTRODUCTION

Klippel-Trenaunay Syndrome (KTS) is an uncommon condition present from birth, which is distinguished by the occurrence of capillary-lymphatic-venous malformation. The incidence rate is estimated to be around 1 in 100,000 individuals.^1^ Somatic mutations in the PIK3CA gene activates PI3K/AKT signaling pathway have been identified as primary causative factors in Klippel-Trenaunay Syndrome (KTS).^2^ Spontaneous extra ring chromosome 18, terminal deletion 2q37.3, and 5:11 balanced translocation have been described in KTS.^3^ It occurs randomly without preference for gender, race, or specific geographical region.^4^ In 1900, Maurice Klippel and Paul Trenaunay documented cases of two patients who exhibited asymmetrical hypertrophy of soft tissues and bones.^5^ We present a case of a 13 years female, with erythema in left upper limb and shoulder.

## CASE REPORT

A thirteen year old female, with erythema in left upper limb and shoulder since birth. At birth, the patient had erythema in the left upper limb and shoulder. The lesion slowly increased in size. Initially, there was compression pain, a slight enlargement in diameter of the left upper limb compared with the right upper limb, and noticeable pain after mild activity, to which no attention was paid at first.

**Figure 1:**
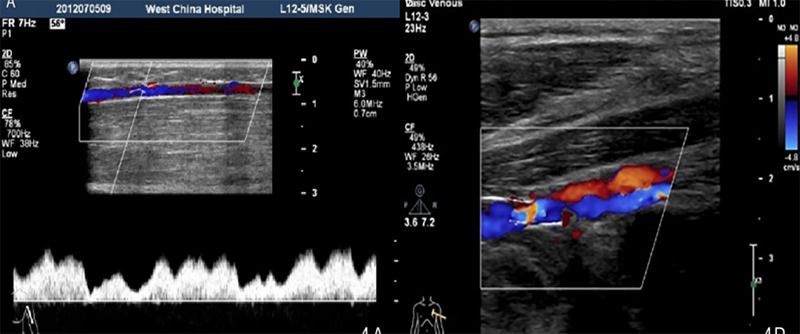
(A) Doppler assessment of pulse wave revealed thinning of the left axillary vein, (B)Left cephalic vein is thicker, draining into axillary vein.

On physical examination, the left upper limb was slightly enlarged than the right. The patient experienced severe pain even on mild physical activity. The pain was negligible at the beginning. The peripheral pulses were palpable, with a normal rate, rhythm, and character. The diameter of the left upper limb was measured and found to be increased. Pain became more pronounced after mild activity. On ultrasound of the upper extremity of the veins, the diameter of the ulnar vein was narrow. The radial vein and the ulnar vein drained into the cephalic vein at the wrist. The diameter of the brachial vein was relatively small, and the lower part of the brachial vein was absent, while the upper part of the brachial vein was about 0.8 mm in diameter at its widest point. The left axillary vein is slightly smaller, approximately 3.0mm (Figure 1). The left basilic vein was absent.

**Figure 2:**
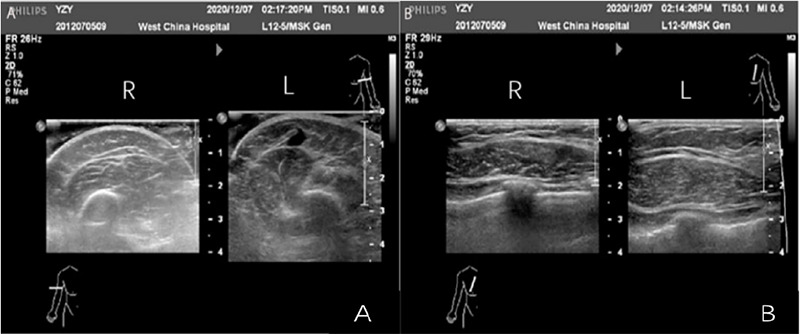
Ultrasound findings of the left sking and muscle of the left hand and chest.

The left cephalic vein was enlarged, measuring approximately 8.5mm where it joined the vein. Swelling and thickening of the affected area of the skin was observed with enlargement of the fingers on the left hand. During a Magnetic resonance imaging of the venous system, there was an increased number of lymph nodes under the subcutaneous fat layer in the left armpit and the left upper arm the largest having approximately 1.0 cm in diameter. Thickening of the skin and subcutaneous layer of the left hand, thickest measuring approximately 7mm (3.5mm on the opposite side) (Figure 2).

**Figure 3:**
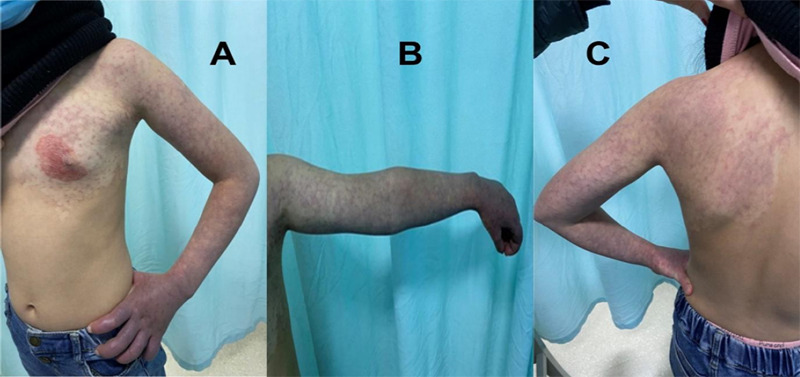
Cutaneous port-wine stain affecting (A) the chest, (B) left arm and (C) back.

Thickening of the subcutaneous and muscle layers of the left chest wall, thickest measuring approximately 23mm (16mm on the opposite side). Thickening of the skin, subcutaneous, and muscle layers of the left upper arm, measuring approximately 41mm (36mm on the opposite side). Thickening of the skin, subcutaneous, and muscle layers of the left forearm, measuring approximately 22mm (19mm on the opposite side). Thickening of the left cephalic vein, with a diameter of approximately 8.5mm at the site of confluence, the thickest part measuring approximately 4mm in the upper arm segment, and approximately 3mm in the forearm. Color Doppler flow imaging (CDFI) and pulse wave (PW) analysis showed normal blood flow velocities and spectral waveforms in the mentioned arterial lumens. Cutaneous port-wine stain affecting the chest, left arm and back was found (Figure 3).

**Figure 4:**
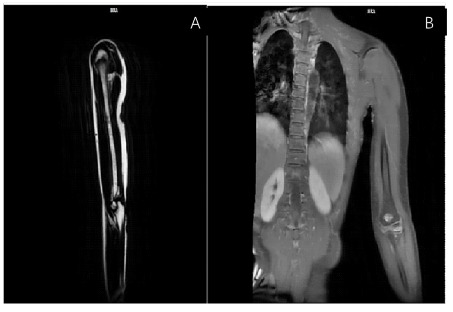
MRI showing the thickness of the subcutaneous area of the chest and left hand.

During MRI examination: The ulnar vein appeared narrower. The radial vein and ulnar vein converged to form a common trunk, which joined the cephalic vein at the wrist. Several tortuous cephalic veins were found around the brachial artery on the left side. The diameter of the brachial vein was normal. The lower segment of the upper arm appeared unclear. The thickest part of the upper arm was approximately 0.8mm. (Figure 4).

The diameter of the left axillary vein was relatively narrow, about 3.0mm. The left basilic vein appeared unclear. The diameter of the left cephalic vein at the site where it joined the axillary vein was approximately 10mm. Blood flow signals were present in the vein lumen. Genetic confirmation of a PIK3CA mutation was not performed due to parental refusal. Based on clinical history and examination, the possible diagnosis could be a rare case of upper extremity erythema in Klippel-Trenaunay syndrome (KTS).

The patient and their family were thoroughly counselled regarding the diagnosis of port-wine stain and its available treatment options, including the benefits, potential risks, and limitations of photodynamic therapy (PDT) and pulsed dye laser (PDL). Despite comprehensive counselling, the family declined all proposed treatments, including photodynamic therapy, due to personal reasons. However, they were made aware of the potential risk associated with untreated PWS and advised on the importance of follow-up to monitor for any progression or complications.

## DISCUSSION

In most cases, tissue hypertrophy is limited to the lower extremities and is unilateral, however, involvement of the upper extremities and, rarely, the trunk has also been reported.^6^ The genetics of KTS is poorly understood, although somatic mutations in the PIK3CA gene are believed to lead to cell proliferation and angiogenesis; somatic mosaicism contributes to the intrapatient and interpatient variability in phenotype.^7^ No clear gender dominance was noted, similar to our investigation where the male-to-female ratio was 9:10.^8^ In our case, tissue hypertrophy was limited to left upper extremities and left anterior chest wall. The diameter of the basilic vein was relatively narrow, and the brachial vein appears thin in the upper segment of the upper arm and absent in the lower segment. The development of collateral veins was poor. The axillary vein was relatively narrow, and the cephalic vein was enlarged near the site where it joined the axillary vein. Left upper limb showed thickening of the subcutaneous layer in the area of erythema, with increased thickness and size of the left hand and fingers. Increased lymph nodes were visualized in the left axilla and the subcutaneous fat layer of the left upper arm, with some slightly enlarged nodes, the largest measuring approximately 1.0cm in short axis.

Klippel-Trenaunay Syndrome (KTS), a rare congenital disorder with diverse clinical presentations, making diagnosis challenging in the absence of genetic testing. Limb size differences often become more evident with growth. A multidisciplinary, patient-centered approach is essential in managing, as it allows for tailored treatment plans that address both the medical complexities and individual preferences of the patient, ultimately improving outcomes and quality of life.
